# Salvianolic acid A as a multifunctional agent ameliorates doxorubicin-induced nephropathy in rats

**DOI:** 10.1038/srep12273

**Published:** 2015-07-21

**Authors:** Hua-Ying Fan, Ming-Yan Yang, Dong Qi, Zuo-Kai Zhang, Lin Zhu, Xiu-Xin Shang-Guan, Ke Liu, Hui Xu, Xin Che

**Affiliations:** 1 School of Pharmacy, Yantai University, 264005 Yantai, Shandong Province, China; 2 Yantai Yu-Huang-Ding Hospital, 264000 Yantai, Shandong, PR China; 3 Shandong Target Drug Research Co. Ltd., Yantai 264005, Shandong Province, China

## Abstract

Nephrotic syndrome (NS) is still a therapeutic challenge. To date there is no ideal treatment. Evidence suggest that multidrug therapy has more effect than monotherapy in amelioration of renal injury. Salvianolic acid A (SAA) is the major active component of *Salviae Miltiorrhizae* Bunge. Previous studies have demonstrated that SAA is a multi-target agent and has various pharmacological activities. The pleiotropic properties of SAA predict its potential in the treatment of NS. The study investigated the effect of SAA on doxorubicin-induced nephropathy. The kidney function related-biochemical changes, hemorheological parameters and oxidative stress status were determined, and histological examination using light and transmission electron microcopies and western blot analysis were also performed. Results revealed that treatment with SAA alleviated histological damages, relieved proteinuria, hypoalbuminemia and hyperlipidemia, reduced oxidative stress, as well as improving hemorheology. Furthermore, SAA restored podocin expression, down-regulated the expression of NF-κB p65 and p-IκBα while up-regulating IκBα protein expression. Overall, as a multifunctional agent, SAA has a favorable renoprotection in doxorubicin-induced nephropathy. The anti-inflammation, antioxidant, amelioration of podocyte injury, improvement of hemorheology and hypolipidemic properties may constituent an important part of its therapeutic effects. All these indicate that SAA is likely to be a promising agent for NS.

Nephrotic syndrome (NS) is a common and multiple disease in the section of nephrology. Its main clinical symptoms include heavy proteinuria, albuminuria, hypoalbuminemia and hyperlipidaemia^1^. Affected patients will progress toward end-stage renal disease if not treated in a timely manner^2^, which threatens patients’ health. Usually, NS is accompanied with severe complications containing hypercoagulation, thrombotic events, metabolism disorders and infections^1^. These factors are potentially lethal^3^. They not only complicate the therapeutic strategies, but also affect the treatment and long-term outcome of this disease^4^.

To date there is no ideal treatment for NS. Hormone and immunosuppressors are the major treatments^5^. In addition to the primary treatments, support therapy constitutes an important part in the treatment of NS, including lipid-lowering therapy, anticoagulation/antiplatelet, and diuretic therapy^5^,^6^. However, these treatments are not always effective to the same degree in all patients, particularly those with refractory nephrotic syndrome, and the serious treatment-related side-effects remain the major concerns^2^,^3^. NS is still a therapeutic challenge. Consequently, there is an urgent need to look for novel therapeutics to attenuate symptoms and complications and offer more therapeutic choices for patients.

The pathogenesis of NS is complex and not entirely clear, which involves multisystem disturbances, namely immunologic, circulatory and metabolic systems. Evidence suggest that oxidative stress, immune injury and inflammation play key roles in the development and progression of NS^2^,^7^,^8^. NF-κB is widely expressed in various tissues and cells, and is related to important pathophysiological processes such as inflammation, immune response and apoptosis. Various factors are associated with the progression of glomerulonephritis, tissue injury in nephrotoxicity and other renal diseases, including IL-2, IL-10, E-selection and MCP-1, which are regulated by NF-κB^9^. Considerable evidence has shown that renal NF-κB activation was present in human renal disease and a variety of experimental models of renal inflammatory disease^10^,^11^,^12^,^13^. Podocyte, a kind of highly differentiated cell that is located at the outer layer of the glomerular basement membrane (GBM), is indispensable in maintaining the integrity of glomerular filtration barrier. It has long been considered that podocyte injury is a significant contributor to proteinuria, and closely related to renal failure. So it is central to prevent podocyte from damage to maintain normal renal function. The mechanisms of podocyte injury have become a research hotspot in recent years. Podocyte protection has been identified as a potentially therapeutic target in the treatment of renal disease^14^. Taken together, the complex pathophysiology of NS indicates that a multi-target agent would offer synergistic beneficial effect on NS, and fully protect kidney from damage. There is evidence that a multidrug therapy consisting of drugs with different mechanisms of action provided more effect than these drugs did alone. These evidence suggest a possible future strategy to lessen renal injury^15^,^16^.

Most traditional Chinese medicine and natural product both have pleiotropic properties, and may have particular therapeutic advantages in the process of NS treatment. As reported previously, the Chinese herbs *Panax Pseudoginseng*, *Radix Astragali* and *Salviae Miltiorrhizae* Bunge have showed favorable renoprotection^17^,^18^,^19^. Thus, looking for candidates from traditional Chinese medicine opens a possible new path for the development of new drugs for the treatment of NS.

Salvianolic acid A (SAA) is one of the major active components of *Salviae Miltiorrhizae* Bunge (also termed as Danshen in China), a versatile Chinese herbal drug. Several pharmacological studies have demonstrated that SAA is a multi-target agent and possesses a variety of pharmacological activities, such as antiplatelet and anti-thrombosis^20^, improvement of microcirculation^20^, anti-inflammation^21^ and antioxidant^22^, and it is the most potent anti-oxidative agent among these compounds extracted from Danshen^22^. Furthermore, in recent years, it has been reported that SAA effectively prevented the development of diabetes and diabetic nephropathy, associated with improvement of lipid metabolism, reduction of proteinuria and oxidative stress^23^,^24^.

Based on the above description, the pharmacological effects of SAA is closely related to the pathophysiology of NS. The pleiotropic properties of SAA predict its potential in the treatment of NS. Thus, we evaluated the effect of SAA in an established model of doxorubicin-induced nephropathy and initially explored its molecular mechanisms.

## Results

### 24 h urinary protein excretion

**Figure 1:**
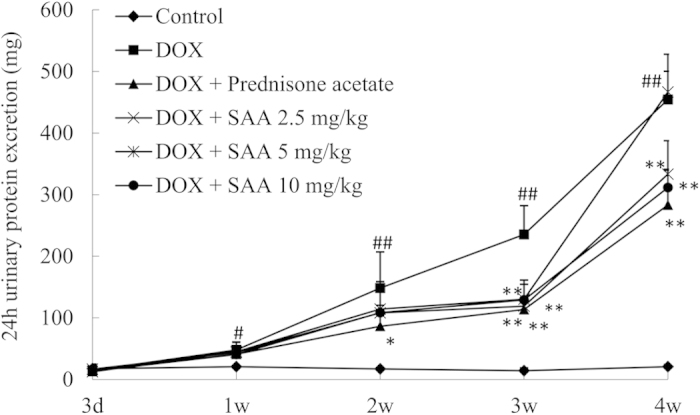
The effect of SAA on urinary protein excretion at each time point. Rats were treated intravenously with SAA at doses of 2.5, 5, and 10 mg/kg or oral administration of prednisone acetate 10 mg/kg after doxorubicin (7.5 mg/kg) injection. Urine was collected for determination of proteinuria on days 3, 7, 14, 21 and 28 after DOX administration. Data are expressed as mean ± SD (n = 5). ^#^*P* < 0.05, ^##^*P* < 0.01 *vs.* Control; ^*^P < 0.05, ^**^P < 0.01 *vs.* DOX alone. SAA, Salvianolic acid A; DOX, Doxorubicin.

As shown in Fig. 1, 24 h urinary protein excretion of rats progressively increased after injection of DOX. On day 7, the urinary protein of DOX-treated rats was significantly higher than that of control rats (*P* = 0.016). Beginning on day 14, the urinary protein of DOX-treated rats rapidly increased (*P* = 0.001). Treatment with prednisone acetate significantly decreased urinary protein at 2, 3, and 4 weeks (*P* = 0.034, *P* = 0.000 and *P* = 0.000, respectively). SAA also decreased urinary protein. The effect was significant at doses of 5 and 10 mg/kg at 3 and 4 weeks (*P* = 0.000, *P* = 0.004 and *P* = 0.001, respectively). SAA 5 mg/kg markedly decreased urinary protein only at 3 weeks (*P* = 0.000), and had no notable effect at 2 and 4 weeks.

### Biochemical Analysis

As indicated in Table 1, intravenous DOX decreased total protein and serum albumin, elevated TG, TC, BUN and SCr levels. Treatment with SAA attenuated the decrease in total protein and serum albumin, reduced the levels of TG, TC, BUN and SCr, and SAA 10 mg/kg showed the best effect relative to the other doses of SAA. Prednisone acetate also exhibited beneficial effect, but its effect on lipid parameters was not comparable to that of SAA.

### Oxidative stress

**Figure 2:**
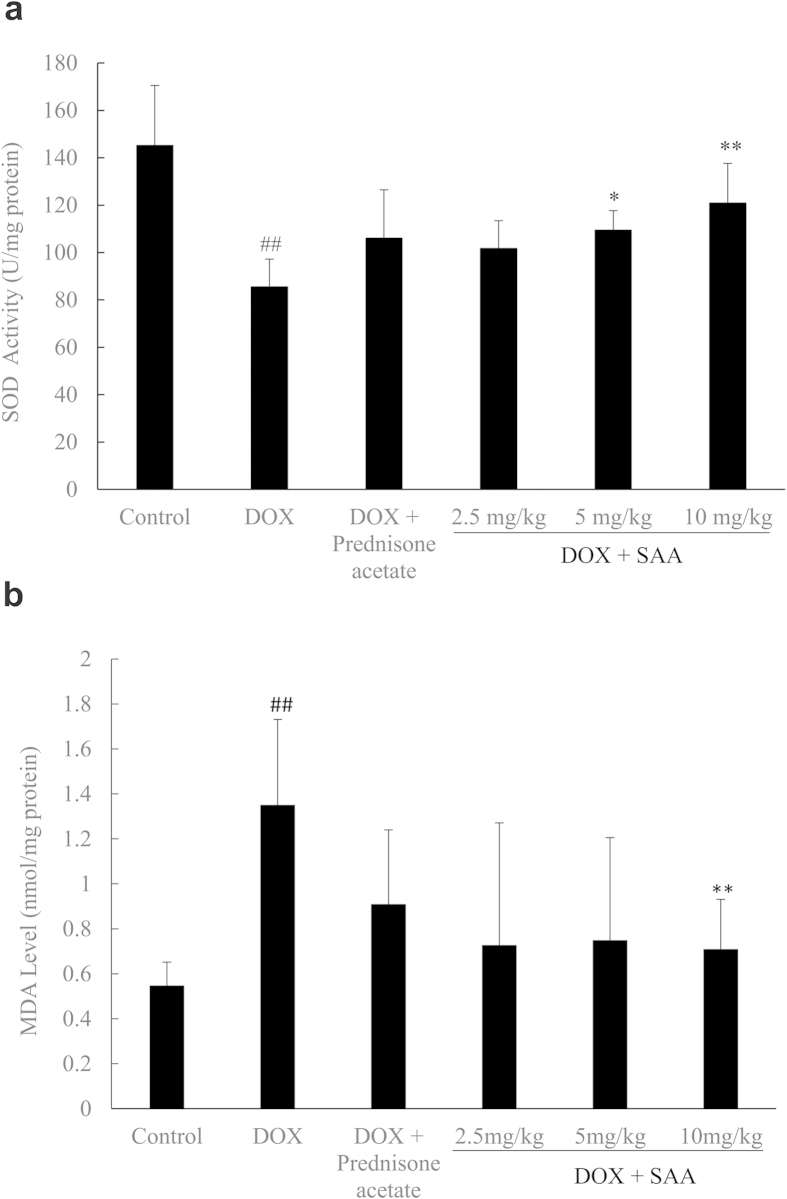
The effects of SAA on superoxide dismutase (SOD) activity and level of malondialdehyde (MDA) in doxorubicin-induced nephropathy rats. **a**: SOD activity, **b**: MDA level. Rats were treated as described in the legend of Fig. 1. At the end of the experiment, rats were sacrificed and renal cortical tissue were collected for determination of SOD activity and MDA level. Data are expressed as mean ± SD (n = 5). ^##^*P* < 0.01 *vs.* Control; ^*^P < 0.05, ^**^P < 0.01 *vs.* DOX alone. Abbreviations: SAA, Salvianolic acid A; DOX, Doxorubicin.

It was demonstrated that SOD activity was lower in DOX-only rats than that in control; meanwhile, increased MDA level was found in nephropathy rats. SAA (2.5, 5, 10 mg/kg) increased SOD activity while decreasing MDA level. The effect was significant at doses of 5 and 10 mg/kg for SOD activity (*P* = 0.026 and *P* = 0.002, respectively), and 10 mg/kg for MDA level (*P* = 0.009). Prednisone acetate had no notable effect on SOD activity and MDA level (*P* = 0.796 and *P* = 0.117, respectively) (Fig. 2a,b).

### Hemorheology

Whole blood viscosity, plasma viscosity and hematocrit considerably elevated in DOX nephropathy rats. SAA substantially improved hemorheologic parameters through decrease of blood viscosity, plasma viscosity and hematocrit (Table 2).

### Light microscopy

**Figure 3:**
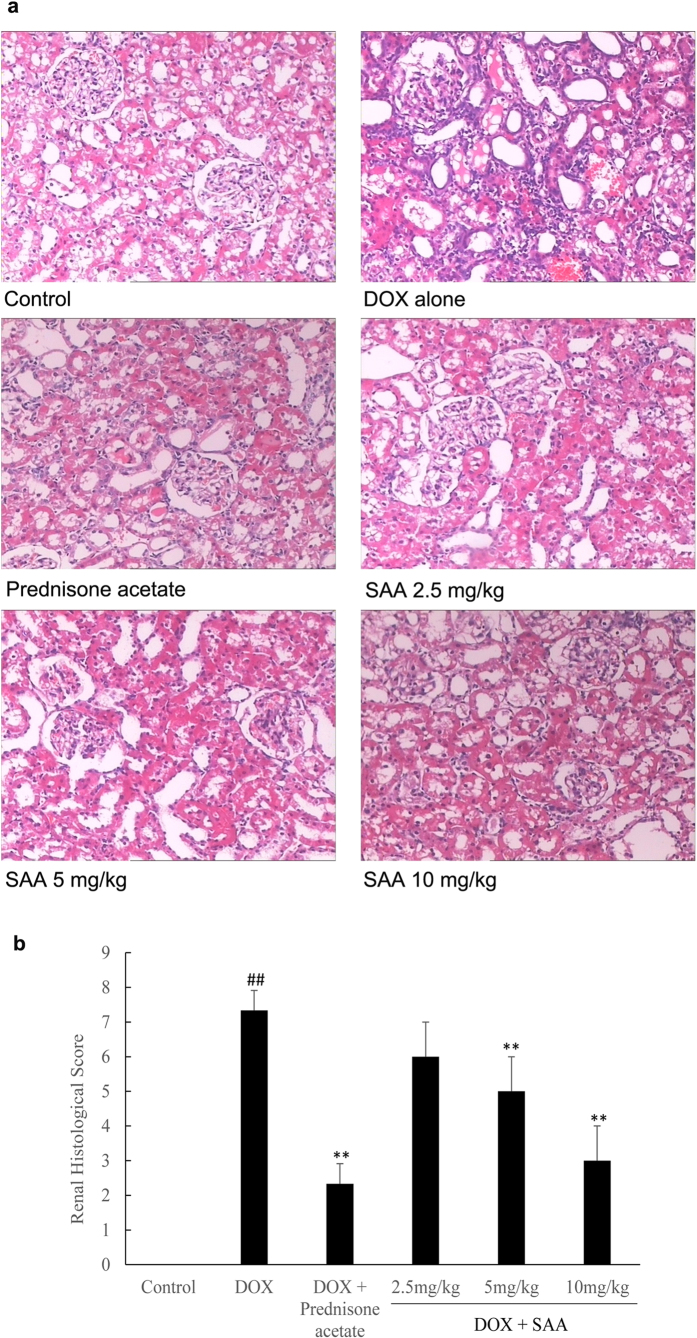
The effect of SAA on renal pathology in doxorubicin-induced nephropathy rats. **a**: Renal histopathologic features; **b**: Pathological scores of renal tissues of each group. Rats were treated as described in the legend of Fig. 1. At the end of the experiment, rats were sacrificed and renal tissue was fixed in 10% formalin. Histopathological analysis was performed in HE-stained sections of renal. Data are expressed as mean ± SD (n = 3). ^##^*P* < 0.01 *vs.* Control; ^**^P < 0.01 *vs.* DOX alone. Original magnification, ×100. Abbreviations: SAA, Salvianolic acid A; DOX, Doxorubicin.

Figure 3 shows representative light microscopy images of renal cortex of each group. There were no histopathological changes in the renal tissue of control rats. Histologic evaluation of the kidney of DOX-treated rats showed marked pathological lesions characterized by glomerular epithelial hyperplasia, tubular dilatation and abundant protein exudation in renal tubular lumen and numerous inflammatory cells infiltrations in renal interstitial. Histopathologic injury scores were significantly elevated (*P* = 0.000). Fortunately, prednisone acetate and SAA with different doses could attenuate the severity of pathological damages in a certain degree, in the form of reduction of inflammatory cells infiltrations and protein cast formation. The pathological scores were correspondingly decreased, and the effect was significant in the groups of SAA 5 and 10 mg/kg and prednisone acetate (*P* = 0.003, *P* = 0.000 and *P* = 0.000, respectively).

### Electron microscopy

**Figure 4:**
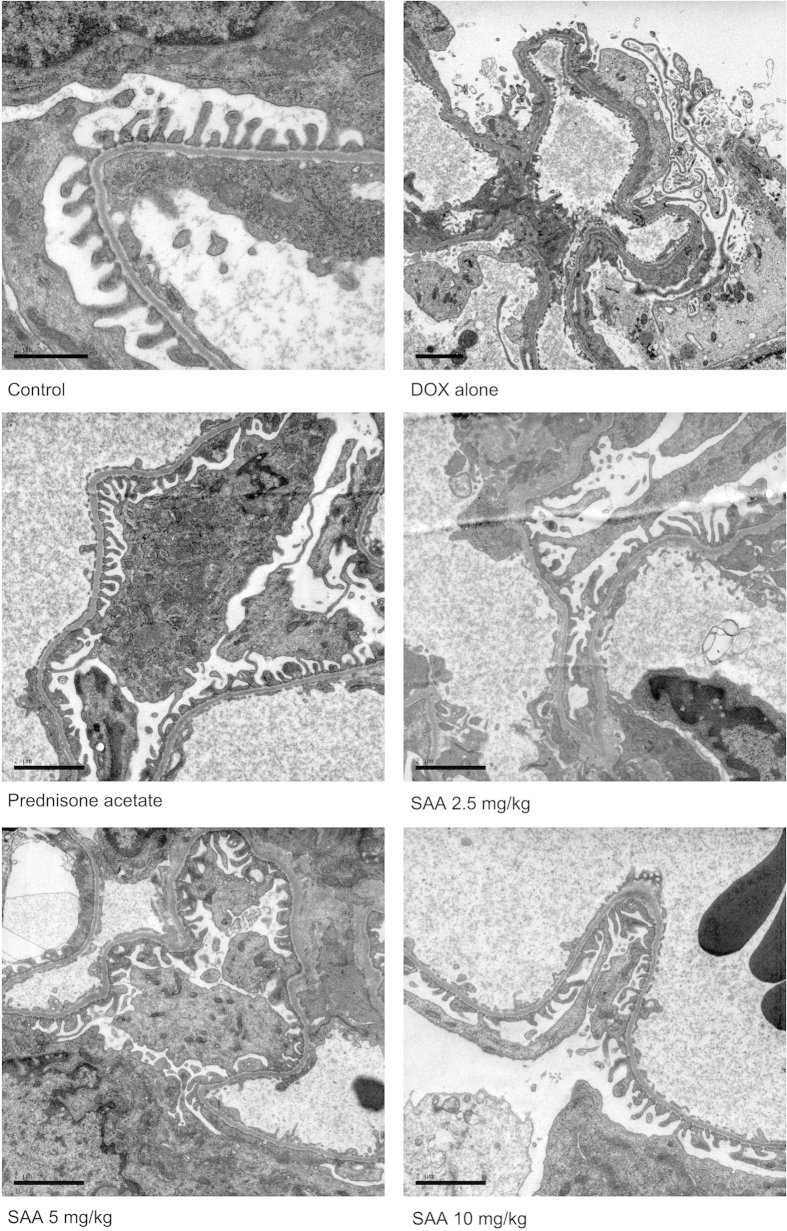
Treatment with SAA ameliorated podocyte foot process effacement in doxorubicin-induced nephropathy rats. Rats were treated as described in the legend of Fig. 1. At the end of the experiment, rats were sacrificed and renal tissue were dissected and fixed for electron microscopic examination. Scale bar, 1 μm or 2 μm. Abbreviations: SAA, Salvianolic acid A; DOX, Doxorubicin.

Transmission electron microscopic examination was performed to analyze the ultrastructural changes in renal tissue. There were no apparent damages as evaluated by intactness of glomerulus and tubules in renal tissues of control rats. The foot processes were clearly observed, and there was no swelling and hypertrophy. In DOX-treated rats, the basal structure of glomerulus was damaged, which manifested as extensive fusion and effacement of foot processes and mild thickening of glomerular basement membrane (GBM). In renal tissues of rats treated with prednisone acetate and SAA, only focal segmental podocyte foot process fusion and effacement were observed. These results suggested that SAA could protect podocyte from damage caused by DOX (Fig. 4).

### Podocin, p- IκBα, IκBα and NF-κB p65 proteins expression

**Figure 5:**
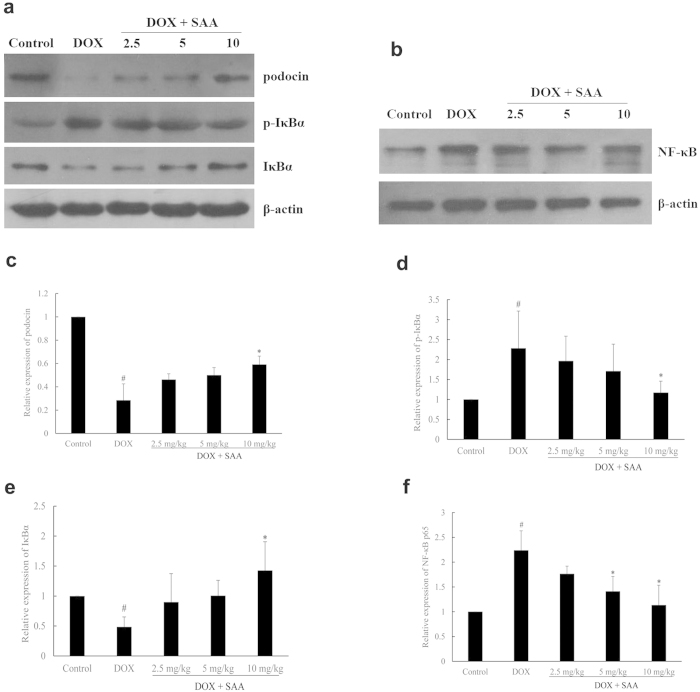
The effect of SAA on podocin, p-IκBα, IκBα and NF-κB p65 protein expressions. **a** and **b**: Protein bands; **c**–**f**: podocin, p-IκBα, IκBα and NF-κB p65 protein expressions relative densities to β-actin. Data are expressed as mean ± SD (n = 3, and n = 4 for NF-κB p65). ^#^*P* < 0.05 *vs*. Control; ^*^P < 0.05 *vs*. DOX alone. Full-length blots are presented in Supplementary Figure 5a and 5b.

As shown in Fig. 5, after DOX administration, p-IκBα and NF-κB p65 protein levels in the DOX-only rats were increased as compared with the control rats, while IκBα expression was decreased, which suggested the activation of NF-κB. In contrast, treatment with SAA down-regulated the expression of p-IκBα and NF-κB p65 while up-regulating the IκBα expression. In addition, a lower expression of podocin, an important podocyte protein involves in the development of proteinuria^25^, was also found in DOX-only rat. SAA significantly recovered the expression level of podocin (*P* = 0.046).

## Discussion

In the current study, we used a single doxorubicin injection method to successfully induce an experimental nephrotic syndrome model, which is characterized by large proteinuria, hyperlipidemia and hypoalbuminemia, increased levels of BUN and SCr (two important indicators reflecting renal function), associated with hyperviscous status and increased oxidative stress. In addition, extensive effacement of podocyte foot processes were also observed in nephrotic rats. But all the features of nephropathy could be lightened by administration of SAA as evidenced by reduction of proteinuria, hypoalbuminemia and glomerular damage, amelioration of renal damage, and improvement of lipid metabolism and hemorheology, as well as reduction of oxidative stress. These results confirm that SAA has a beneficial effect on DOX nephropathy. Furthermore, the effect of SAA appeared to be superior to that of prednisone acetate in terms of lipid-lowering and improvement of hemorheology.

Venous and arterial thromboembolism are considered to be the most severe complications of NS, which affect the treatment and outcome of NS. Hypercoagulability status was considered to contribute to this thrombophilic phenomenon^26^. Patients with nephrotic syndrome usually suffer from hyperviscosity, local intravascular coagulation and altered hemorheology. It is thought that high blood viscosity may attributed to hypoproteinemia and hyperlipidemia. Increased blood viscosity and decreased blood flow would alter hemorheology in the renal microcirculation and tend to induce thrombosis and eventually worsen renal damage. It was reported that treatment to correct altered hemorheology with antiplatelet and anticoagulants has successfully prevented renal disease progression^27^,^28^,^29^. In China, the drugs with characteristics of promoting blood circulation and removing blood stasis are widely used in clinical, and offer beneficial effects to patients through improving renal function and attenuating complications^17^,^19^,^30^. We have previously demonstrated that SAA effectively improved microcirculation and ameliorated blood stasis^20^. And then, we evaluated the effect of SAA on hemorheology in nephrotic rats. The present findings revealed that blood viscosity and hematocrit were increased in nephrotic rats. SAA markedly improved hemorheologic parameters through decreasing blood viscosity and hematocrit, thereby promoting circulation and improving renal blood flow.

Oxidative stress is one of the significant mechanisms in the pathogenesis of renal injury, which is produced by excessive generation of reactive oxygen species (ROS) or inefficient antioxidant defenses^7^,^31^. Various studies have confirmed that oxidative stress and abnormality in antioxidative system are present in nephrotic patients^7^,^32^,^33^,^34^. SOD and MDA are two ideal markers of oxidative stress and tissue injury. SOD is one of the important enzymatic antioxidants and has an effect on modifying systemic antioxidant status to prevent the harmful effects of ROS. MDA is a product of polyunsaturated fatty acid peroxidation as a measurement of lipid oxidation^35^. It has been reported that treatment with SOD or antioxidant supplementation markedly reduced proteinuria and attenuated renal damage possibly by inhibiting lipoprotein oxidation and reducing oxidative stress^36^,^37^,^38^. As claimed in the introduction section, SAA possesses potent antioxidant activity, thus we tested the effect of SAA on oxidative stress. The present data showed that lower SOD activity and increased MDA level were found in nephrotic rats as compared with the control rats. Treatment with SAA reduced MDA accumulation and increased SOD activity. This could be likely associated with the capacity of improving antioxidant defense of SAA.

The beneficial effect of SAA was further illustrated by histological evaluation using light and transmission electron microcopies. Histopathologic examination showed that glomerular epithelial hyperplasia, tubular dilatation, abundant protein exudation in renal tubular lumen and numerous inflammatory cells infiltrations were present in renal tissue of nephrotic rats. In addition, extensive fusion and effacement of podocyte foot processes could be also observed. Treatment with SAA decreased the severity of pathologic damage, reduced extent of podocyte foot effacement. It is known that podocytes are critical in maintaining the normal filtration barrier of glomerulus and podocyte slit diaphragm (SD) plays a major role within it. Nephrin and podocin are protein components of SD, involving in regulating normal renal function^39^. Podocin plays an important role in maintaining the structure and function of SD. Research has shown that reduced expression of podocin lead to the development of proteinuria and renal disease^25^. Thus, restoration of the key slit diaphragm proteins expression and prevention of podocyte injury are potentially effective intervention for nephropathy. In the present study, treatment of rats with SAA restored the expression of podocin in renal tissue of rats with DOX-induced nephropathy. Taken together with the results of reduced extent of podocyte foot effacement by treatment with SAA, we may conclude that SAA could maintain normal morphology of podocyte and the glomerular filtration barrier. Although we have demonstrated the podocyte protection of SAA, additional mechanisms underlying the therapeutic effect of SAA on podocyte in kidney need to be further investigated. In addition to podocin, other proteins including CD2-associated protein, nephrin, densin and basement membrane proteins are also associated with podocyte injury, as well as Wnt and Notch signaling pathways^14^,^40^.

As noted earlier, NF-κB as a transcription factor plays a central role in the regulation of a large number of genes involved in the progression of renal disease. NF-κB is activated in podocytes and persistent proteinuria and peroxides are stimulants of NF-κB. The activation of NF-κB is regulated by IκB protein and IκB kinases (IKKs). When inactive, the NF-κB dimer associates with IκB proteins, the best-studied and most important of which is IκBα^41^. In response to several stimuli, the IKKs are activated and phosphorylate the inhibitory IκBα protein, resulting in the dissociation of IκBα from NF-κB. The uncovered nuclear localization signals will then cause the activation of NF-κB proteins^41^. Inhibition of the NF-κB activation reduces proteinuria and ameliorates renal injury^9^,^13^,^42^. Currently, most drugs used in clinical practice for renal disease, such as antioxidants, glucocorticoids, angiotensin converting enzyme (ACE) inhibitors and statins, both directly or indirectly inhibit NF-κB activation^10^. In addition, it has been reported that the mechanisms of anti-inflammatory effects of SAA may be attributable to its modulation of NF-κB dependent inflammatory pathways via IKKβ inhibition^21^. In the present study, we examined whether SAA modulated the activation of NF-κB in renal tissue of nephrotic rats. Our data showed that treatment with SAA decreased the expression of NF-κB p65 and p-IκBα while increasing the expression of IκBα. Therefore, decreased NF-κB expression and increased IκBα protein together with decreased p-IκBα expression, might be the potential mechanism involved in the renoprotection of SAA.

In conclusion, our results suggest that SAA has multiple therapeutic activities and can partially protect against DOX-induced nephropathy in rats. The anti-inflammation, antioxidant, inhibition of podocyte injury, improvement of hemorheology and hypolipidemic properties may constituent an important part of its therapeutic effects. The molecular mechanism may involve the inhibition of NF-κB activation and regulation of podocin expression. These novel findings provide the pharmacological foundation for the further development of SAA for the treatment of NS.

## Methods

### Animals

Male Sprague-Dawley (SD) rats (weight, 180–200 g) were purchased from Vital River Laboratory Animal Technology Co., Ltd. (certificate No.11400700039133). All animals were allowed to acclimate for at least 1 week at a temperature of 24 ± 1 °C and humidity of 55% ± 5%. All rats were housed in cages with food and tap water ad libitum. All experiments were performed in accordance with relevant guidelines and regulations approved by the Experimental Animal Research Committee of Yantai University.

### Materials and regents

SAA was provided by Shandong Target Drug Research Co. Ltd (Shandong, China). Prednisone acetate was the product of Zhejiang Xianju Pharmaceutical Co. Ltd. (Zhejiang, China). Doxorubicin hydrochloride for injection was produced by Shenzhen Main Luck Pharmaceuticals Inc. (Shenzhen, China). The antibodies used in this study were anti-NF-κB p65 (ab16502, Abcam), anti-inhibitor of NF-κB (IκB) α (ab32135, Abcam), anti-phosphorylation-IκBα (p-IκBα, Ser-36, ab133462, Abcam) and anti-podocin (sc-21009, Santa Cruz Biotechnology, Inc.). Goat anti-rabbit IgG was also the product of Abcam. BCA protein assay kit, anti-β-actin antibody, HRP-labeled goat anti-mouse IgG (H+L) and lipid peroxidation malonaldehyde (MDA) assay kit were obtained from Beyotime Institute of Biotechnology (Jiangsu, China). Superoxide dismutase (SOD) detection kit was purchased from Nanjing Jiancheng Bioengineering Institute (Nanjing, China).

### Animal model and treatment protocol

Thirty male Sprague-Dawley (SD) rats were randomly divided into six groups of five animals each: control, doxorubicin (DOX) alone, DOX plus prednisone acetate (10 mg/kg, ig), DOX plus SAA (2.5, 5, 10 mg/kg, iv). Doxorubicin (7.5 mg/kg) were given intravenously once to rats to induced kidney injury as previously described^43^. Rats in control group were injected with normal saline instead. Seven days (on day 8) after DOX injection, rats were administered with SAA (dissolved in 5% glucose solution) and prednisone acetate, and continued for 21 days. Animals in control and DOX-only group were administered with the same volume of glucose solution.

### Biochemical Analysis and Hemorheology

On days 3, 7, 14, 21 and 28 after DOX injection, rats in each group were put into metabolic cages, and 24-hour urine samples were collected for measurement of urinary protein. On days 29, these rats were sacrificed with intraperitoneal injection of chloral hydrate (350 mg/kg). Blood were collected from aorta abdominalis, and four milliliters of blood were anti-coagulated with heparin. Hemorheological parameters of hematocrit (Hct), plasma viscosity (PV) and whole blood viscosity (WBC) including high shear rate, middle shear rate and low shear rate were determined by routine laboratory assays. The rest of the blood were centrifuged for 15 min at 3000 rpm. The serum was isolated and the levels of serum albumin (ALB), total cholesterol (TC), triglyceride (TG), serum creatinine (Scr) and blood urea nitrogen (BUN) were measured by Automatic Biochemical Analyzer. Twenty-four hours of urinary protein excretion and serum total protein were measured by BCA protein assay kit.

### Kidney tissue preparation and Histological examination

Before collection of blood, the two kidneys were rapidly removed. The left kidney was cut into two parts. One part of the kidney was fixed in 10% formalin, embedded in paraffin and stained with hematoxylin and eosin (H and E), and assessed under light microscopy. Sections were examined by a qualified and blinded pathologist to evaluate the degree of pathological changes based on glomerular epithelial hyperplasia, tubular dilatation and protein cast formation, and inflammatory cells infiltrations. These were scored from 0 to 3 (normal to severe). The total score for each renal sample was calculated. Another part of the kidney was used for electron microscopic examination. Small blocks of kidneys were fixed in 3% glutaraldehyde, postfixed in 2% osmium tetroxide, dehydrated in graded ethanol and acetone, and then embedded in epoxy resin. Ultrathin section were stained with lead citrate and uranyl acetate and examined using an electron microscopy (JEM-1400, Japan). The renal cortex from right kidney was stored at −80 °C and thawed just before use.

### Determination of SOD activity and MDA level

The renal cortex was homogenized on ice in PBS buffer. The supernatants were prepared by centrifugation at 12000 rpm for 10 min and collected, and then total protein concentration was measured using a BCA kit. The supernatants were then used for the determination of activity of SOD and MDA level. The process of assay was performed according to the manufacturers’ instructions. The final results were expressed as U/mg protein for SOD activity and μmol/mg protein for MDA level.

### Western blot analysis

Nuclear and cytoplasmic extracts were prepared using a nuclear and cytoplasmic protein extraction kit (Beyotime, China). Briefly, renal cortex (approximately 80 mg) were homogenized in cytoplasmic extraction buffer. Tissue homogenates were rapidly lysed by vortexing. The homogenate was centrifuged at 12000 rpm for 10 min at 4 °C and proteins were collected from the supernatant. The pellets were re-suspended in 50 μL of nuclear protein extraction buffer, lysed by ultrasound in an ice water bath, and then centrifuged to yield the nuclear fraction. Protein concentration was measured with a BCA protein assay kit. Protein samples (80–100 μg) were separated using 10% SDS–PAGE gels and transferred onto PVDF membranes. The membranes were blocked with 5% skim milk in Tris-buffered saline with Tween 20 for 2 h at room temperature and then incubated with primary antibodies to anti-podocin (1:200 dilution), anti-NF-κB p65, anti-IκBα, or anti-p-IκBα (1:4000–1:10000 dilution) overnight at 4 °C. Then, the membrane was washed 4 times for 10 min each and incubated with a horseradish peroxidase-conjugated anti-rabbit IgG antibody. Protein bands were detected using an enhanced chemiluminescence detection kit (Beyotime Institute of Biotechnology) and visualized by exposure to photographic film. The protein bands were visualized by exposure to photographic film. Densitometric analysis of protein bands was performed using Quantity One v4.4.0.36 analyzer software (BIO-RAD).

### Statistical analysis

Statistical analyses were performed using SPSS software 17.0 for Windows. All results are expressed as mean ± standard deviation (SD). Quantitative data were tested for homogeneity of variance. If the variance was homogeneous, one-way ANOVA followed by the least significant difference (LSD) test was used. Comparisons between groups of nonparametric data were made using the Kruskal-Wallis test followed by the Mann-Whitney *U* test. *P* < 0.05 was considered significant.

## Additional Information

**How to cite this article**: Fan, H.-Y. *et al.* Salvianolic acid A as a multifunctional agent ameliorates doxorubicin-induced nephropathy in rats. *Sci. Rep.* **5**, 12273; doi: 10.1038/srep12273 (2015).

## Supplementary Material

Supplementary Information

## Figures and Tables

**Table 1:** The effect of SAA on biochemical parameters in doxorubicin-induced nephropathy rats.

**Group**	**ALB (g/L)**	**Total protein (g/L)**	**TG (mmol/L)**	**TC (mmol/L)**	**BUN (mmol/L)**	**SCr (umol/L)**
Control	35.00 ± 2.55	65.96 ± 15.66	0.99 ± 0.14	1.13 ± 0.23	7.23 ± 0.54	51.33 ± 2.66
Doxorubicin	21.10 ± 1.15^##^	14.29 ± 8.85^##^	9.10 ± 1.68^#^	7.55 ± 1.71^#^	15.30 ± 2.89^##^	81.70 ± 8.15^##^
Doxorubicin + Prednisone acetate	25.67 ± 2.87^*^	37.59 ± 14.77^**^	7.48 ± 0.31	4.75 ± 0.68^*^	9.60 ± 1.14^**^	60.27 ± 7.42^**^
Doxorubicin + SAA 2.5 mg/kg	23.18 ± 3.32	20.44 ± 10.10	6.62 ± 4.14	4.29 ± 1.37^*^	11.61 ± 3.41^*^	73.30 ± 9.56
Doxorubicin + SAA 5 mg/kg	25.33 ± 3.15^*^	27.51 ± 2.83	4.68 ± 3.89	4.18 ± 1.88^*^	9.50 ± 1.25^**^	69.70 ± 2.45^*^
Doxorubicin + SAA 10 mg/kg	25.95 ± 2.14^*^	32.03 ± 7.59^*^	3.57 ± 1.73^*^	4.46 ± 0.79^*^	9.73 ± 1.89^**^	64.48 ± 6.17^**^

Data are expressed as mean ± SD (n = 5).

^#^*P* < 0.05, ^##^*P* < 0.01 *vs.* Control; ^*^P < 0.05, ^**^P < 0.01 *vs.* DOX alone.

Abbreviations: ALB, Albumin; TG, Triglycerides; TC, Total Cholesterol; BUN, Blood urea nitrogen; SCr, Serum creatinine; SAA, Salvianolic acid A; DOX, Doxorubicin.

**Table 2:** The effect of SAA on hemorheologic parameters in doxorubicin-induced nephropathy rats.

**Group**	**WBV (mPa.s)**			**PV (mPa.s)**	**Hct (%)**
	**Low shear rate**	**Middle shear rate**	**High shear rate**		
Control	24.82 ± 4.97	6.52 ± 1.33	4.77 ± 0.83	1.67 ± 0.40	0.42 ± 0.02
Doxorubicin	39.66 ± 9.07^##^	8.86 ± 0.96^##^	6.38 ± 0.47^##^	2.24 ± 0.51^#^	0.45 ± 0.02^##^
Doxorubicin + Prednisone acetate	31.96 ± 7.13	7.78 ± 1.01^*^	5.44 ± 1.25^*^	1.79 ± 0.23	0.45 ± 0.03
Doxorubicin + SAA 2.5 mg/kg	25.82 ± 3.05^**^	7.52 ± 0.83^*^	5.30 ± 0.62^*^	1.51 ± 0.08^**^	0.44 ± 0.03
Doxorubicin + SAA 5 mg/kg	24.47 ± 1.24^**^	6.85 ± 1.40^**^	5.48 ± 1.14^*^	1.77 ± 0.15	0.43 ± 0.03
Doxorubicin + SAA 10 mg/kg	23.28 ± 3.17^**^	6.86 ± 0.78^**^	5.24 ± 0.51^*^	1.53 ± 0.06^**^	0.42 ± 0.02^*^

Data are expressed as mean ± SD (n = 5).

^#^*P* < 0.05, ^##^*P* < 0.01 *vs.* Control; ^*^P < 0.05, ^**^P < 0.01 *vs.* DOX alone.

Abbreviations: WBV, Whole blood viscosity; PV, Plasma viscosity; Hct, Hematocrit; SAA, Salvianolic acid A; DOX, Doxorubicin.
