# Supplementary material for: Salvianolic acid A as a multifunctional agent ameliorates doxorubicin-induced nephropathy in rats
Source: Sci Rep. 2015 Jul 21;5:12273. doi: 10.1038/srep12273 (PMC4508635; doi:10.1038/srep12273)
Supplement: Supplementary Information [file srep12273-s1.pdf]

Supplementary Data

Title: Salvianolic acid A as a multifunctional agent ameliorates doxorubicin-induced nephropathy in rats

Authors: Hua-Ying Fan, Ming-Yan Yang, Dong Qi, Zuo-Kai Zhang, Lin Zhu, Xiu-Xin Shang-Guan, Ke Liu, Hui Xu, Xin Che

Figure 5 a

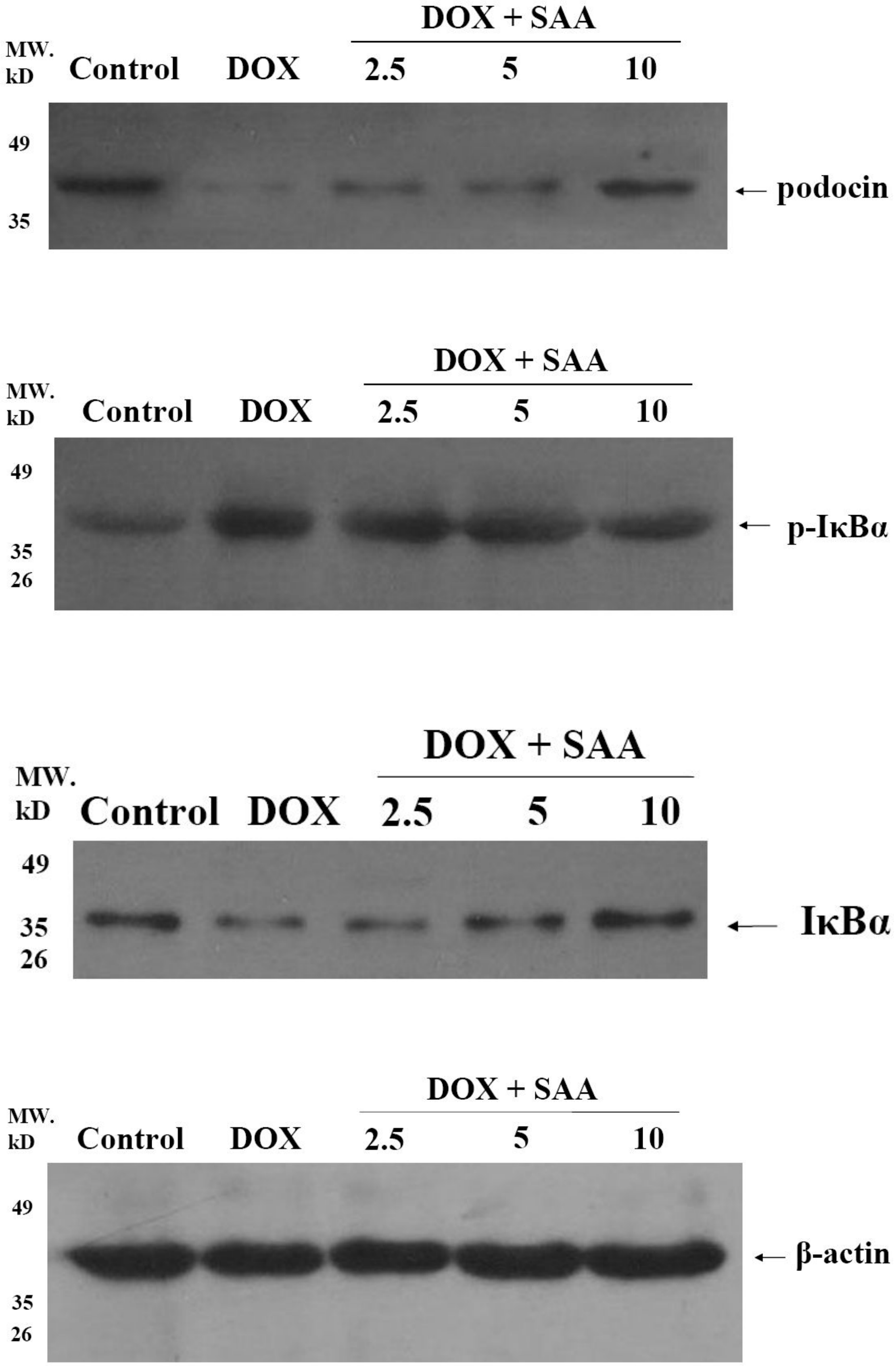
DOX + SAA
MW.
kD
Control
DOX
2.5
5
10
49
35
← podocin
DOX + SAA
MW.
kD
Control
DOX
2.5
5
10
49
35
26
← p-IκBα
DOX + SAA
MW.
kD
Control
DOX
2.5
5
10
49
35
26
← IκBα
DOX + SAA
MW.
kD
Control
DOX
2.5
5
10
49
35
26
← β-actin

Figure 5 b

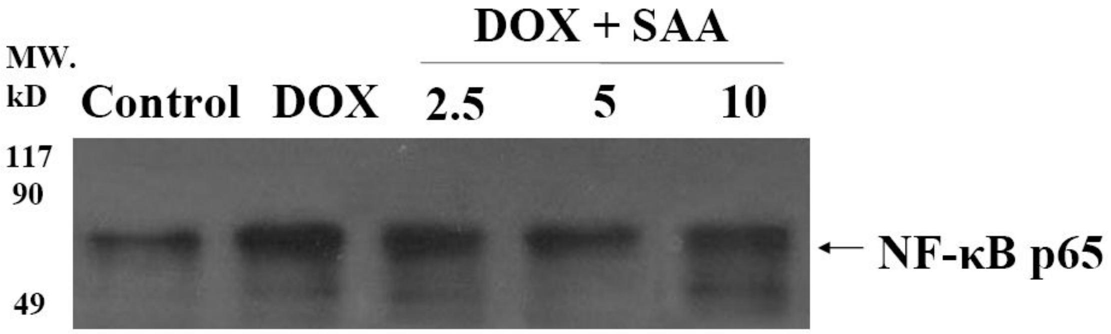

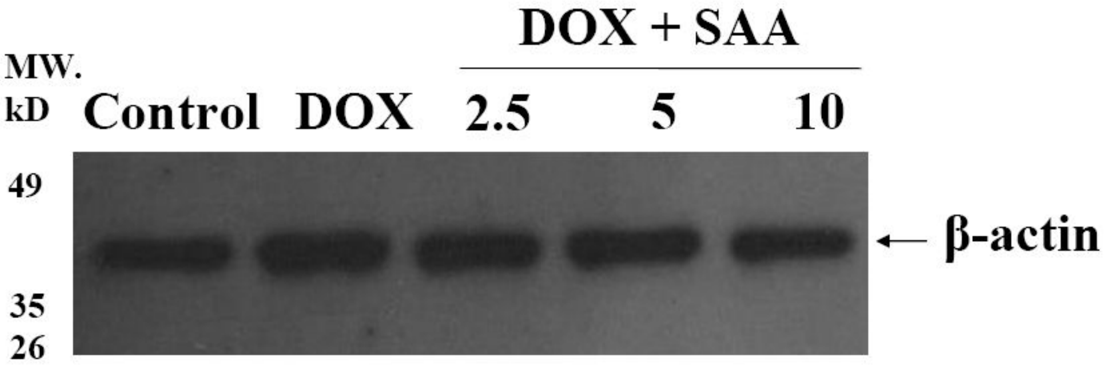

Figure Legend

Figure 5. The effect of SAA on podocin, p-IκBα, IκBα and NF-κB p65 protein expressions. a and b: Protein bands. Abbreviations: SAA, Salvianolic acid A; DOX, Doxorubicin.
